# Patients, Doctors, and Chatbots

**DOI:** 10.2196/50869

**Published:** 2024-01-04

**Authors:** Thomas C Erren

**Affiliations:** 1 Institute and Policlinic for Occupational Medicine, Environmental Medicine and Prevention Research University Hospital of Cologne University of Cologne Köln (Zollstock) Germany

**Keywords:** chatbot, ChatGPT, medical advice, ethics, patients, doctors

## Abstract

Medical advice is key to the relationship between doctor and patient. The question I will address is “how may chatbots affect the interaction between patients and doctors in regards to medical advice?” I describe what lies ahead when using chatbots and identify questions galore for the daily work of doctors. I conclude with a gloomy outlook, expectations for the urgently needed ethical discourse, and a hope in relation to humans and machines.

## Introduction

While I strive to provide accurate and helpful information, I am not a substitute for medical advice or professional judgment, and it’s always important for patients and healthcare providers to work together to develop a personalized treatment plan that takes into account a patient’s individual needs and circumstances.

ChatGPT, 2023

Medical advice (MA) is key to the relationship between doctor and patient. The question I will address is “how may chatbots affect the interaction between patients and doctors in regards to medical advice?” To this end, I shall consider—and go beyond—what was recently outlined regarding MA in “A Conversation With ChatGPT” [1].

Advances in artificial intelligence (AI) and chatbots are changing the world, including medicine [2-4]. ChatGPT is a generative pretrained transformer model based on GPT-3 from OpenAI. Based on word correlations in its 175 billion–parameter database, ChatGPT floods us with meaningful but also nonsensical information.

Concerning the interaction between patients, doctors, and chatbots, I describe what lies ahead when using chatbots and identify many questions for the daily work of doctors. I conclude with a gloomy outlook, expectations for urgently needed ethical discourse [5,6], and a hope in relation to humans and machines [3,7].

## Weighing ChatGPT’s Quote

How ChatGPT describes its role [1]—“I am not a substitute for medical advice”—should be a fact. Doctors, as the only authoritative providers of professional MA, must always be in the driver’s seat. Chatbots have the potential to help with the task of contributing general information to an information chain. Importantly, doctors need to review and question all AI output and see if and how it contributes to a patient’s understanding and fits within MA. Depending on the expectations and hopes that ChatGPT raises in patients, this task could become an unprecedented challenge.

With their up-to-date knowledge and medical experience and expertise, doctors need to integrate personal, specific, and general information into their comprehensive MA to the patients. Chatbots are limited to general information stored in databases. Concerningly, ChatGPT invents facts, called a hallucination in AI [3]. Moreover, ChatGPT can produce nonsensical or “bullshit” [8] information, nicely worded and seemingly justified but disregarding truth and facts—disconcertingly, we do not readily know how often and when ChatGPT offers “bullshit” or nonsense responses.

## The Daily Work of Doctors: Question Galore

Nevertheless, ChatGPT will be used by many simply because it is there and seemingly easy and, importantly, free to use.

Is it, therefore, likely that we can do without chatbots? No, because society will not abandon ChatGPT or other advanced chatbot tools [3]. The sooner we understand chatbot information for patients, the better. Realistically, ChatGPT is just the tip of an AI iceberg. The “Godfather of AI” [9] Hinton and OpenAI’s chief executive officer Altman [10] have warned forcefully about the speed, impact, and inevitability of AI developments.

Doctors routinely deal with both informed and misinformed patients, fuelled by online health searches (eg, “Dr Google” [11]). Indeed, the internet has become the starting point for many to ask questions about health, disrupting traditional doctor-patient relationships [12] and leading to potential harm from online misinformation [11]. Importantly, neither patients nor doctors should give away too much information when using AI. Even if MA could get better with more details, can we know if this information is being used beyond MA? Indeed, to what extent may creating MA be used as an AI Trojan horse to extract information for other purposes, including business benefits? Which biases go into AI-based medical information, for instance, through training data that neither represent the ethnicity nor the financial options of diverse patients? That medically advanced AI may become expensive raises questions of equity: who will have access to these technologies?

What knowledge do doctors need to understand medical AI advice? How can AI-based medical information be used [13], and how do you deal with medical information that AI cannot explain [14]? Could doctors working with chatbot-provided diagnoses and AI-recommended treatments miss the true picture and become overreliant on AI? Who is liable when doctors use AI medical information, and to come full circle, who is liable when they do not [2,15]? Could there come a time when not considering AI such as ChatGPT constitutes less than adequate advice and nonstandard care [15]? Doctors should ask their liability insurer how (ie, under what conditions) and to what extent the insurer covers the use, or nonuse, of AI in practice [15].

Key orientation for interactions between patients, doctors, and chatbots regarding MA can come from physicians’ professional organizations and the US Food and Drug Administration. Similar to practice guidelines [15], recommendations and guardrails for practice-specific medical information via chatbots may have to be developed.

## A Gloomy Outlook, Expectations From Much-Needed Ethical Discourse, and a Hope in Relation to Humans and Machines

That ChatGPT “strive(s) to provide accurate and helpful information” [1] has a stale empirical aftertaste. In fact, according to OpenAI, advanced AI [16] will make reviewing chatbot information even more difficult. GPT-4 (eg, in Microsoft Bing and ChatGPT Plus), with 571 times as many learned parameters as GPT-3, has “learned” to deliver incorrect work more convincingly than earlier models. Such mistakes will pose severe problems even if “[ChatGPT] admits these when challenged” [1].

PubMed-listed comparisons between GPT-3 and GPT-4 suggest that the latter may provide more accurate patient information in nuclear medicine [17]. Another study suggested that both free and paid versions of ChatGPT risk providing misleading responses when used without expert MA [18]. Chatbot medical information written at a college reading level suggested that such AI devices may be used supplementarily but not as a primary source for medical information [19], emphasizing the doctor’s key role in MA. More research is needed on MA in numerous medical fields and settings, for numerous applications, and for various populations.

Overall, when AI experts at the University of California, Berkeley explored and discussed the implications of ChatGPT and AI and future challenges in the spring of 2023, there was an explicit call for more ethical considerations [6,20]. Priority safety measures include strict regulations for patient privacy and ethical practices [21]. While the questions above are not exhaustive, it is time to systematically answer them regarding MA and the unavoidable interaction of patients, doctors, and chatbots.

Ultimately, we can only hope that the boundaries between humans and machines [3] will never become so blurred that patients cannot distinguish the MA of a human doctor from the general information provided by ChatGPT [22] or other AI.

## Abbreviations

AI: artificial intelligence
MA: medical advice

## References

[1] Eysenbach G (2023). The role of ChatGPT, generative language models, and artificial intelligence in medical education: a conversation with ChatGPT and a call for papers. JMIR Med Educ.

[2] Haupt CE, Marks M (2023). AI-generated medical advice-GPT and beyond. JAMA.

[3] Shaw D, Morfeld P, Erren T (2023). The (mis)use of ChatGPT in science and education: Turing, Djerassi, "athletics" & ethics. EMBO Rep.

[4] Coghlan S, Leins K, Sheldrick S, Cheong M, Gooding P, D'Alfonso S (2023). To chat or bot to chat: ethical issues with using chatbots in mental health. Digit Health.

[5] Akerson M, Andazola M, Moore A, DeCamp M (2023). More than just a pretty face? Nudging and bias in chatbots. Ann Intern Med.

[6] Erren TC, Lewis P, Shaw DM (2023). Brave (in a) new world: an ethical perspective on chatbots for medical advice. Front Public Health.

[7] Turing AM (1950). I.—Computing machinery and intelligence. Mind.

[8] Frankfurt HG (2005). On Bullshit.

[9] Metz C (2023). ‘The Godfather of A.I.’ leaves google and warns of danger ahead. The New York Times.

[10] Fung B (2023). Mr. ChatGPT goes to Washington: OpenAI CEO Sam Altman testifies before Congress on AI risks. CNN.

[11] Hyman I (2020). The risks of consulting Dr. Google. Psychology Today.

[12] Freckelton I (2020). Internet disruptions in the doctor-patient relationship. Med Law Rev.

[13] Zhu Y, Wang R, Pu C (2022). "I am chatbot, your virtual mental health adviser." What drives citizens' satisfaction and continuance intention toward mental health chatbots during the COVID-19 pandemic? An empirical study in China. Digit Health.

[14] Wang F, Kaushal R, Khullar D (2020). Should health care demand interpretable artificial intelligence or accept "Black Box" medicine?. Ann Intern Med.

[15] Price WN, Gerke S, Cohen IG (2019). Potential liability for physicians using artificial intelligence. JAMA.

[16] (2023). GPT-4 technical report. OpenAI.

[17] Currie G, Robbie S, Tually P (2023). ChatGPT and patient information in nuclear medicine: GPT-3.5 versus GPT-4. J Nucl Med Technol.

[18] Deiana G, Dettori M, Arghittu A, Azara A, Gabutti G, Castiglia P (2023). Artificial intelligence and public health: evaluating ChatGPT responses to vaccination myths and misconceptions. Vaccines (Basel).

[19] Pan A, Musheyev D, Bockelman D, Loeb S, Kabarriti AE (2023). Assessment of artificial intelligence chatbot responses to top searched queries about cancer. JAMA Oncol.

[20] Manke K (2023). AI lectures at Berkeley to explore possibilities, implications of ChatGPT. Berkeley News.

[21] The Lancet (2023). AI in medicine: creating a safe and equitable future. Lancet.

[22] Nov O, Singh N, Mann D (2023). Putting ChatGPT's medical advice to the (Turing) test: survey study. JMIR Med Educ.

